# Integrated Analytical System for Clinical Single‐Cell Analysis

**DOI:** 10.1002/advs.202200415

**Published:** 2022-05-04

**Authors:** Hannah M. Peterson, Lip Ket Chin, Yoshi Iwamoto, Juhyun Oh, Jonathan C. T. Carlson, Hakho Lee, Hyungsoon Im, Ralph Weissleder

**Affiliations:** ^1^ Center for Systems Biology Massachusetts General Hospital Boston MA 02114 USA; ^2^ Cancer Center Massachusetts General Hospital Boston MA 02114 USA; ^3^ Department of Radiology Massachusetts General Hospital Boston MA 02114 USA; ^4^ Department of Systems Biology Harvard Medical School Boston MA 02115 USA

**Keywords:** biopsy, cancer, deep profiling, diagnostic

## Abstract

High‐dimensional analyses of cancers can potentially be used to better define cancer subtypes, analyze the complex tumor microenvironment, and perform cancer cell pathway analyses for drug trials. Unfortunately, integrated systems that allow such analyses in serial fine needle aspirates within a day or at point‐of‐care currently do not exist. To achieve this, an integrated immunofluorescence single‐cell analyzer (i2SCAN) for deep profiling of directly harvested cells is developed. By combining a novel cellular imaging system, highly cyclable bioorthogonal FAST antibody panels, and integrated computational analysis, it is shown that same‐day analysis is possible in thousands of harvested cells. It is demonstrated that the i2SCAN approach allows comprehensive analysis of breast cancer samples obtained by fine needle aspiration or core tissues. The method is a rapid, robust, and low‐cost solution to high‐dimensional analysis of scant clinical specimens.

## Graphical abstract

An inexpensive integrated immunofluorescence single‐cell analyzer (i2SCAN) is developed for deep profiling of harvested cells. By combining a novel cellular imaging system, highly cyclable bioorthogonal FAST antibody panels, and integrated computational analysis, Authors show that same‐day analysis is possible in thousands of harvested cells. The method is a rapid, robust, and low‐cost solution to high‐dimensional analysis of scant clinical specimens.

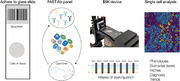

## 1 Introduction

Phenotypic classification of tumors is essential in clinical decision making, unraveling the complexity of human tumor microenvironment and determining the presence of drug‐able targets. Clinically, this is currently achieved by obtaining tumor tissue through surgical excision or image‐guided biopsies with subsequent immunohistochemical staining to yield data on a relatively small subset of markers per sample. Rapid advances in molecular oncology, however, have raised unique unmet needs: i) establishing higher‐dimensional biomarker panels rather than relying on a few chosen biomarkers, ii) performing analysis at higher throughput in an automated fashion and in short periods of time (same day), iii) substituting invasive core biopsies with lower morbidity fine needle aspirates (FNAs), and iv) performing temporal analyses of the tumor microenvironment in patients undergoing treatments. If possible, such an approach could have significant impacts on patient care in medical centers and point‐of‐care settings across the globe. Unfortunately, widely used conventional immunohistochemistry approaches are associated with technical limitations such as low throughput, requirements for multiple sections to process different biomarkers, frequent paucity of cytological sample materials resulting in nondiagnostic cases and interobserver variability.^[^ ^1^ ^]^

A number of highly multiplexed immunofluorescence techniques have emerged,^[^ ^1^, ^2^, ^3^, ^4^, ^5^ ^]^ but many of them face similar constraints of long turn‐around times, computational complexity, high cost, and incompatibility with cytology specimens due to harsh cycling conditions. As a result, the methods are primarily used as research tools to study cell composition, cellular function, and cell–cell interactions. Similarly, low‐cost and integrated microscopes have been described,^[^ ^6^ ^]^ but they have yet to be adapted for multiplexed fluorescence analysis of clinical samples.

We have been addressing the above needs by developing automated image cytometry systems^[^ ^7^ ^]^ that can be used in remote settings where the access to resources and expert help is restricted.^[^ ^8^ ^]^ FNAs, yielding cells rather than tissue, are ideally suited in such environments given the very low morbidity, simplicity of sample acquisition, and chromogenic staining. Up to now, such samples still required expert cytological interpretation, and immunostaining may not be available in remote locations, especially for scant samples. We have been thus interested in developing more advanced automated systems to analyze both FNA and tissue core samples, i.e., the clinically most common sample types. To further improve the diagnostic accuracy and utility, we were also interested in combining the device with ultrafast multiplexing capabilities (FAST), so that cyclic imaging could be performed within a few hours rather than days to weeks. Recent advances in chemistry^[^ ^9^, ^10^, ^11^, ^12^ ^]^ are beginning to enable such workflows^[^ ^13^ ^]^ but have not been implemented for breast cancer.

Here, we report an inexpensive, robust, multichannel single‐cell imaging system (i2SCAN: integrated immunofluorescence single‐cell analyzer) with integrated analysis. We combined i2SCAN with FAST cyclic imaging to measure >12 biomarkers in scant cells and tissues. We implemented such a system and workflow through iterative improvements of design and based on practical insights from field testing. The design requirements included no moving filters to speed up acquisition and durability, integrated operation to minimize manual intervention, and computational analyses yielding clinically actionable results. Using breast cancer as a model system, we show that i2SCAN overcomes many of the previous technical hurdles and expands profiling capabilities in the clinic.

## 2 Results

### 2.1 i2SCAN Enables Rapid Acquisition of Whole Slide Datasets

**Figure 1:**
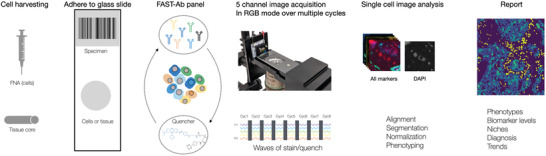
Overview of the i2SCAN workflow. Cells are harvested by fine needle aspiration, fixed and adhered to glass slides. Alternatively, tissue fragments from core biopsies are processed for FFPE tissue sections and deposited on glass slides. FAST‐antibody panels are used for rapid cell staining, imaging, and subsequent quenching (<10 min for FNAs). This cycle is iterated until the entire marker panel is imaged. Each multichannel image acquisition is typically completed in <1 min. Cells are then segmented, and marker expressions are computationally derived.

Three major impediments exist to enable accurate and same‐day molecular diagnosis of solid cancers: i) a considerable fraction of cytological specimens processed by conventional means are nondiagnostic;^[^ ^14^, ^15^ ^]^ ii) highly trained specialists, who are needed to review samples, are often lacking in low‐ and middle‐income countries and point‐of‐care settings;^[^ ^16^ ^]^ and iii) the sample processing is complex and long, involving tissue embedding, sectioning, and sequential re‐staining with different antibodies. Conversely, it has been shown that deep molecular profiling of cells obtained by FNA enables molecular diagnoses^[^ ^11^, ^17^ ^]^ but such technology has not yet been adapted to routine clinical workflows or in remote settings. We thus set out to develop such a workflow encompassing i) a low‐cost integrated device; ii) unique reagents and staining protocols for rapid deep multiplexing of cellular samples; and iii) computational analyses to facilitate interpretation (**Figure** **1**). Such a workflow could enhance clinical practice, decrease the number of nondiagnostic samples, and minimize the time delay to the initiation of therapy (Figure S1, Supporting Information).

### 2.2 i2SCAN System Design and Characterization

**Figure 2:**
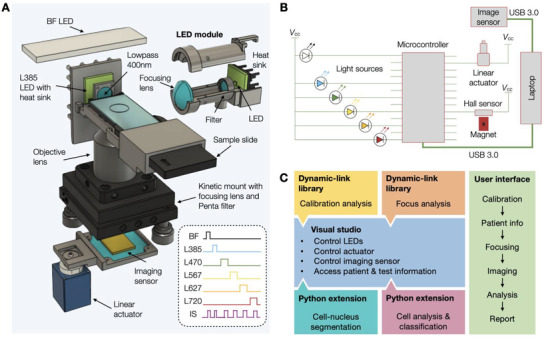
i2SCAN imaging system. A) 3D rendering showing the lateral light path of an LED to illuminate the sample in such a way that the imaging sensor in not flooded. LEDs are mounted on heat sinks to stabilize output power. A total of six LEDs are used. A linear actuator is used for auto‐focusing. B) Schematic diagram of a custom‐designed microcontroller for sequential illumination of the sample through each LED and synchronization with the image sensor. C) A standalone software package was developed with a user‐friendly interface to: i) perform daily calibration of the system; ii) control the LEDs, linear actuator, and imaging sensor; iii) perform auto‐focusing and image capturing; iv) perform image analysis for cell‐nucleus segmentation and classification.

**Figure** **2** and Figure S2 in the Supporting Information summarize key components of the standalone i2SCAN system. The optics design addressed some of the limitations of a previous analytical system^[^ ^8^, ^18^ ^]^ which enabled only three‐color imaging without the ability to perform deeper multiplexing required for accurate diagnoses. We purposely designed a new system without a moving excitation and emission filter turret. This approach improved durability, minimized service needs, decreased system cost, and sped up image acquisition. We used a five‐channel penta filter and configured a lateral illumination light path (Figure 2A). Instead of expensive lasers or multispectral light sources, we used multiple light‐emitting diodes (LEDs); each LED was cooled by a heat sink to stabilize light output. The image was acquired by a color CMOS (complementary metal oxide semiconductor) imager which was mounted on a linear actuator for auto‐focusing. We chose a color imager over a monochromatic one to perform spectral fingerprinting as well as compensate for channel bleed through, allowing us to profile single cells in six colors. The overall system operation was controlled by an embedded microcontroller (Figure 2B) that synchronized LED on‐offs with image acquisition. Images were acquired with only one LED turned on at any given time. Figure 2C details the computational aspects of image acquisition and analysis. A standalone software package was developed with a user‐friendly interface to perform calibration, auto‐focusing, image capture, and subsequent analysis (see below and Figure S3, Supporting Information).

### 2.3 Characterization of i2SCAN Optics

**Figure 3:**
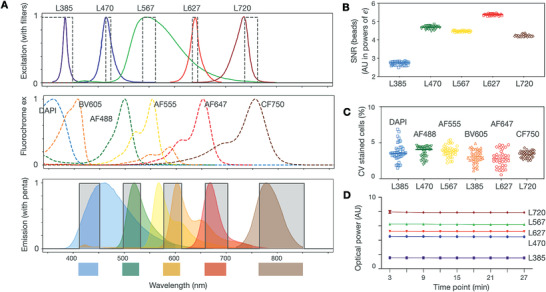
Spectral characteristics. A) Summary of spectral characteristics of i) the five LEDs with excitation filters (to limit broader LED spectra), ii) fluorochrome excitation, and iii) fluorochrome emission with a penta emission filter. B) SNR of beads stained with DAPI (blue), AF488 (green), AF555 (yellow), AF647 (red), and CF750 (brown). Note the uniformly high SNR across LED/filter/fluorochrome combinations. C) The CV of cells stained by six different fluorochromes. D) Repeat measurements of light output through different LEDs. Note the narrow CV.

**Figure** **3** shows i2SCAN spectral characteristics and performance of image acquisition (see Figure S4 in the Supporting Information for additional detail). The system had a white LED for bright‐field imaging and five color LEDs for fluorescent imaging (L385nm, L470nm, L567nm, L627nm, L720nm). Each color LED was coupled with an in‐line bandpass filter to narrow the excitation wavelength (Figure 3A and Figure S4, Supporting Information). This set‐up allowed us to use commonly used fluorochromes such as DAPI (4′,6‐diamidino‐2‐phenylindole), BV605, AF488, AF555, AF647, and CF750. The penta filter aligned with the emission wavelengths of each of these fluorochromes.

We first tested the system with fluorescent beads and fluorochrome‐stained cells. To rule out the interference from bleed through, we used beads and cells of a single fluorescent color. The bead signal‐to‐noise ratio (SNR) was high and uniform, reaching >200 for some of the indocyanine fluorochromes (Figure 3B). We also performed similar experiments with stained cells and directly compared SNR values between the i2SCAN system and a commercial microscope equipped with LED light sources and multibandpass filters. Across all fluorescent channels, i2SCAN displayed higher SNR than the commercial system (L470/AF488: i2SCAN 86.3 vs microscope 65.6; L567/AF555: i2SCAN 73.0 vs microscope 46.0; L627/AF647: i2SCAN 214.5 vs microscope 168.5; L720/CF750: i2SCAN 7.9 vs microscope 3.9). This can be attributed to the side‐illumination scheme in i2SCAN, which minimized the entrance of stray excitation light into an imager. The coefficient of variance (CV) for stained cells in an entire field of view was narrow with mean values around 4% (Figure 3C). Finally, we monitored the optical output through each LED and confirmed that it was within specification over prolonged use with the CV less than 0.5% (Figure 3D).

### 2.4 Automated RGB Fingerprinting Yields Unmixed, High‐Quality Image Analysis

Fluorescence imaging of cells stained with multiple fluorochromes invariably leads to fluorescence bleed through into adjacent channels given the broad emission spectra of organic rhodamine/cyanine dyes.^[^ ^19^, ^20^ ^]^ To remove such artifacts (beyond the use of filters and lateral illumination) in i2SCAN images, we developed an RGB fingerprinting method by analyzing pixel RGB outputs of each fluorochrome and compensated for bleed through signals.

**Figure 4:**
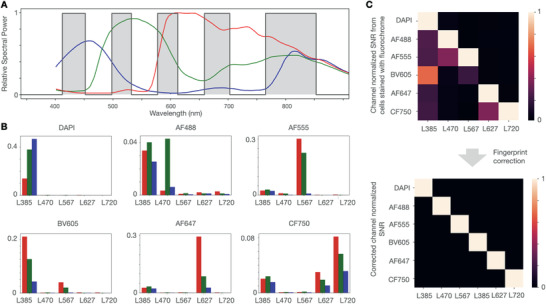
Fingerprinting analysis. A) The relative sensitivities of RGB channels are superimposed on the light spectrum. The gray bars represent the five emission gaps that the CMOS camera sees through a penta filter. B) Experiment using BT474 cells stained with the indicated fluorochromes and showing signal detected by the camera pixels when each of the LED is turned on sequentially. For example, for cells stained with AF555, the camera detects strong red and green signals when L567 is turned on. However, the camera also detects a smaller amount of RGB signals when L385 is turned on (mostly autofluorescence), a small signal with L470 (crosstalk), and negligible signal when L627 or L720 are turned on. Different fluorochromes show distinct color signature (fingerprint) with different LED and RGB sensors. C) Raw SNR data from cells stained with different fluorochromes and illuminated with different LEDs (top). Without correction, there is up to 60% of bleed through into adjacent channels. After fingerprint correction (bottom), the signals are clearly separated.

**Figure** **4**A overlaps the emission spectral windows of the penta filter with the relative sensitivity of RGB sensors in the i2SCAN imager. The RGB sensors create a unique signature for each fluorochrome that is used for spectral unmixing. Figure 4B details the experimental measurements of RGB signals for a given LED and fluorochrome. The highest bleed through was observed with L470/AF488 (into the DAPI channel), L385/BV605 (into the AF555 channel), and with L720/CF750 (into the AF647 and DAPI channels). Given the different ratios on R, G, and B channels, we used this information to correct images for bleed through.

This analysis resulted in high contrast and bleed through free images (Figure 4C). To further test the algorithm, we also performed cell staining experiments. Figure S5 in the Supporting Information shows raw and corrected cell images stained with the above fluorochromes, demonstrating that the spectral unmixing resulted in high contrast cellular images.

### 2.5 Multiplexed Analysis in Single‐Cell Suspensions

Tumor cells can be clinically harvested from palpable mass lesions by image‐guided FNAs. This minimally invasive procedure uses 22–25G needles to obtain single cells or cell clusters for subsequent analysis. An average of ≈2 mg of material or ≈1000 cells (range 100–5000) can be obtained from a single needle pass. Because the materials can be of such low cellularity, the rate of nondiagnostic specimens can be as high as 30%,^[^ ^21^, ^22^ ^]^ even with expert sampling and processing. To determine whether i2SCAN could be used for multiplexed analyses of single cells, we first processed cell lines whose marker presence is established and can be independently validated by flow cytometry.

**Figure 5:**
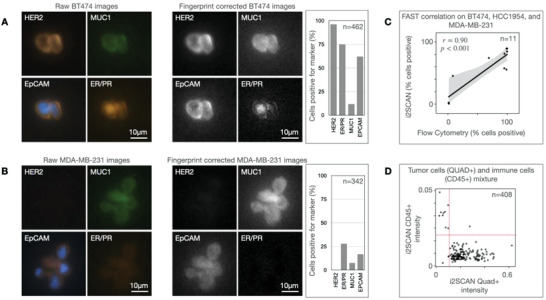
Automated single‐cell analysis. To determine whether single cells could be stained with FAST probes and analyzed through fingerprinting, we examined BT474 (HER2+/ER+/PR+) and MD‐MB‐231 cells (triple negative breast cancer). A) Representative examples of BT747 stained for the markers shown (left) and automated analysis of biomarker expression after fingerprint correction (right). Note the high levels of HER2 and ER/PR. B) Examples of triple‐negative MD‐MB‐231 cells stained for the same markers and showing low levels of HER2 and ER/PR, but positive signals from cancer markers (EpCAM, MUC1). C) Correlation between i2SCAN measurements and flow cytometry. Note the good correlation (Pearson r = 0.90). D) i2SCAN analysis of tumor cell/PBMC mixture displayed CD45 versus QUAD (HER2/EpCAM/MUC1/EGFR). Note the clean separation (Raw images in panels A and B are brightness and contrast adjusted).

**Figure** **5** shows a representative example of triple‐positive (HER2+/ER+/PR+) BT474 breast cancer cells, stained and analyzed by i2SCAN. Virtually all cells were positive for HER2 (human epidermal growth factor receptor 2), ER (estrogen receptor), PR (progesterone receptor), EGFR (epidermal growth factor receptor), and EpCAM (epithelial cell adhesion molecule). In contrast, the triple negative (HER2‐/ER‐/PR‐) MDA MB231 cells showed negligible fluorescent signal for HER2, ER, and PR, but were positive for cancer markers (EGFR (not shown) and MUC1 and EpCAM). Figure 5C shows that there was good correlation between i2SCAN analysis and flow cytometry (Pearson *r* = 0.90). In an analysis of a tumor cell/immune cell mixture, a clean separation between tumor (HER2+/EpCAM+/MUC1+/EGFR+) and immune cells (CD45+) was observed (Figure 5D). Once the above approaches were validated in cell lines, we proceeded to processing primary human samples (**Table** **1**). Staining was performed with the FAST cycling method (Figure S6, Supporting Information), which is compatible with cellular harvests.

**Table 1:** Samples studied. All primary samples were de‐identified. PBMC: peripheral blood mononuclear cells

Sample	Type	Malignancy	N	Age (range)
Cell line	BT474	Yes	1	60
	HCC1954	Yes	1	61
	MCF‐7	Yes	1	69
	MDA‐MB‐231	Yes	1	51
	Benign breast	No	4	Undisclosed
Primary isolate	PBMC	No	3	Undisclosed
FFPE	Mixed human breast cancers	Yes	26	34–79
	Normal breast tissue	No	3	19–21
**Total**			40	

### 2.6 Multifactorial Analysis of Tissue Sections Provides High Accuracy

**Figure 6:**
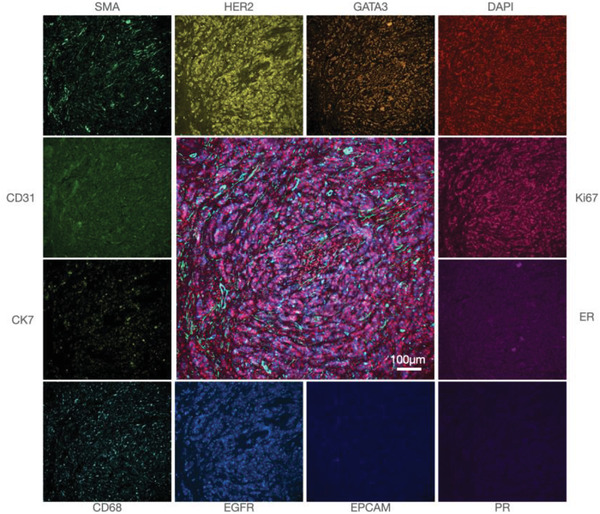
Single marker analysis. Breast cancer FFPE section stained for 14 biomarkers using the FAST cycling method. 12 representative markers are shown in monochromatic color from a conventional microscope and the center image is a fusion of GATA3, DAPI, Ki67, SMA, and EGFR. Images were acquired in four cycles of staining and quenching.

We next proceeded to analyze tissue samples such as those obtained during surgical resection or core biopsies. Because tissues are embedded in paraffin and sectioned, additional optimization and validation steps were necessary for the FAST‐FFPE (formalin‐fixed paraffin‐embedded) cycling technique (Figure S7, Supporting Information). Compared to images acquired with a conventional microscope taken at a similar magnification (10x objective, 1728 × 1728 pixels), the i2SCAN has a larger field of view (2448 × 2048 pixels) with a similar pixel resolution (≈0.6 µm pixel^−1^). For additional comparison, a commercial whole slide scanner had a large field of view (51 840 × 21 760 pixels) and a resolution of 0.5 µm pixel^−1^. As shown in Figure S7 in the Supporting Information, the image quality is comparable among the three methods. Based on iterative analyses, we changed some of the antibodies for better performance in tissues (**Tables** **2**, **3**, **4**). Once optimized, we proceeded to analyze tissue samples from patients. **Figure** **6** provides a summary of the representative raw images from a panel of 14 biomarkers imaged in breast cancer specimens.

**Table 2:** Antibodies for flow and direct single cell staining

	Marker	Tumor subtype/cell type	Vendor	Cat #	Clone	Host	Cell line
1	EpCAM	Tumor cells	Biolegend	324202	9C4	Mouse	BT474
2	EGFR	Tumor cells	Abcam	ab30	EGFR1	Mouse	MDA‐MB‐231
3	HER2	Tumor cells	BioRad	MCA1788	ICR55	Rat	BT474, HCC‐1954
4	HER2	Tumor cells	Biolegend	324402	24D2	Mouse	BT474
5	ER (ESR1)	Tumor cells	CST	13258S	D6R2W	Rabbit	BT474
6	PR (PGR)	Tumor cells	CST	8757S	D8Q2J	Rabbit	BT474
7	MUC1	Tumor cells	Fitzgerald	10‐M93A	10‐M93A	Mouse	MDA‐MB‐231
8	CD45	Immune cells	CST	13917	D9M8I	Rabbit	PBMC
9	IgG Control	N/A	CST	3900	DA1E	Rabbit	N/A
10	IgG Control	N/A	Biolegend	400501	RTK2758	Rat	N/A
11	IgG Control	N/A	Biolegend	400102	MOPC‐21	Mouse	N/A
12	IgG Control	N/A	Biolegend	400302	MPC‐11	Mouse	N/A

**Table 3:** FAST antibodies for staining of paraffin‐embedded tissue sections (FFPE)

	Marker	Tumor subtype/cell type	Vendor	Cat #	Clone	Host	Fluor	Cell line
1	EpCAM	Tumor cells	R&D Systems	AF960	Polyclonal	Goat	AF488	A431
2	EGFR	Tumor cells	R&D Systems	AF231	Polyclonal	Goat	AF488	A431
3	HER2	Tumor cells	CST	45332BC	29D8	Rabbit	AF488	BT474
4	ER (ESR1)	Tumor cells	Abcam	ab282199	SP1	Rabbit	AF555	MCF 7
5	PR (PGR)	Tumor cells	R&D Systems	AF5415	Polyclonal	Sheep	AF647	T‐47D
6	Ki67	Tumor cells	Abcam	ab15580	Polyclonal	Rabbit	AF647	A431
7	CK7	Tumor cells	BioLegend	601602	W16155A	Rat	AF647	RT4
8	MUC1	Tumor cells	BioLegend	355602	16A	Mouse	CF750	U‐2 OS
9	TROP2	Tumor cells	Abcam	ab214488	EPR20043	Rabbit	AF647	BT474
10	GATA3	Tumor cells	CST	5852BF	D13C9	Rabbit	AF555	A431
11	CD45	Immune cells	CST	47937SF	D9M8I	Rabbit	AF555	PBMC
12	CD68	Immune cells	CST	916104	KP1	Mouse	AF647	PBMC
13	CD31	Immune cells	CST	85873SF	89C2	Mouse	AF488	HUVEC
14	CD34	Immune cells	Bio‐Rad	MCA547G	QBEND/10	Mouse	BV605	HUVEC
15	CD8	Immune cells	CST	85336BF	D8A8Y	Rabbit	AF647	PBMC
16	SMA	Host cells	Thermo Fisher	14‐9760‐82	1A4	Mouse	AF555	A431

**Table 4:** Labeling kits

	Marker	Vendor	Cat #
1	Mix‐n Stain CF750 Antibody Labeling Kit, 1x (5–20 ug)	Biotium	92284
2	Brilliant Violet 605 Goat anti‐mouse IgG (clone: Poly4053)	BioLegend	405327

**Figure 7:**
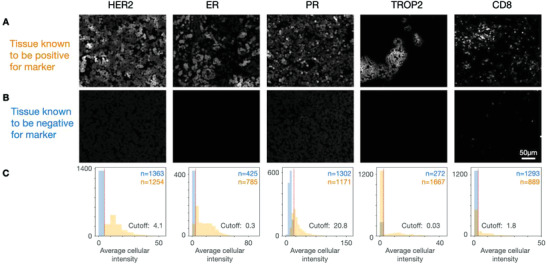
i2SCAN of biomarkers in FFPE sections. Tissue microarrays representing 24 patients with pathology proven breast cancer and variable expression levels of HER2, ER, and PR were analyzed by i2SCAN. Shown are representative examples for biomarker positive and negative cases. A) Synthetic images of representative cases positive for the biomarker shown and which was corroborated with IHC, FISH, and other testing. B) Synthetic images of representative cases confirmed negative for the biomarker shown. C) Summary plots of signal in segmented tumor cells. The threshold for a given marker was determined in representative samples (with and without controls).

Setting appropriate thresholds for biomarker analysis is essential in performing cell‐by‐cell‐based analyses. We determined the thresholds for marker positivity by single‐cell analysis and correlation to daughter sections processed for immunohistochemistry, similarly as done in other immunological validation studies.^[^ ^23^ ^]^ Based on the threshold values, i2SCAN was then used to analyze tissue for marker presence, degree of positivity, and spatial arrangement. **Figure** **7** summarizes representative examples of thresholds for key therapeutic targets (HER2, ER, PR, TROP2, CD8).

**Figure 8:**
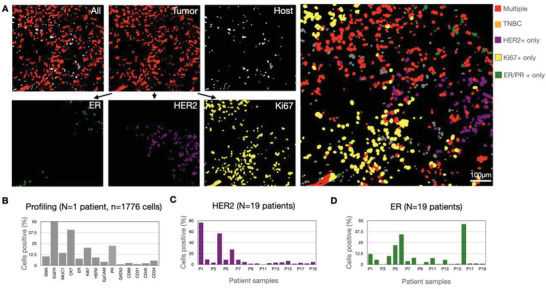
Analysis of cellular biomarkers in patient cohort. A) Synthetic images derived from FFPE tissue section. Note the exclusive separation of tumor and host cells and the ability to analyze individual cells for markers. Top right: Mosaic of individual cancer cells that express multiple markers (red; positive for HER2, ER, PR, and/or Ki67), Ki67 only (yellow), HER2 only (purple), ER/PR only (green), or no HER2/ER/PR/Ki67 (orange). B) Summary of biomarker expression in one patient sample. A total of 14 markers were analyzed. C) Summary of HER2 expression across patient cohort (n = 19 patients). D) Summary of ER expression across patient cohort (n = 19 patients).

We next determined whether the i2SCAN method could be used to analyze a cohort of breast cancer tissues. FFPE samples from 19 patients were obtained and processed via FAST staining and i2SCAN automated analysis. In a first step, we computationally separated host from tumor cells at the single‐cell level. **Figure** **8**A shows a representative example of such analysis where host cells are clearly separated from tumor cells in fibrous septa. Tumor cell analysis was subsequently performed to determine the expression level of each protein of interest in a given cell. This allowed us to display HER2, ER, or multiparameter cellular maps of the tumor environment. These analyses also allowed patient (Figure 8B) and cohort wide (Figure 8C,D) analyses. In summary, the above experiments demonstrate that the i2SCAN approach allows comprehensive analysis of breast cancer samples obtained by fine needle aspiration or core tissue sections.

## 3 Discussion

In this study, we describe i2SCAN, an integrated combined workflow enabling the rapid analysis of co‐expression of dozens of proteins in thousands of single cells. This is important because single‐cell protein analysis has not been widely adapted for clinical use for a number of reasons. Although a considerable number of different cycling technologies have been published for FFPE sections,^[^ ^1^, ^24^ ^]^ these tools are mostly used in research given their long staining and destaining times, high cost, and lack of reimbursement. Furthermore, the issue of adapting high‐dimensional analysis to scant cytological samples has generally not been solved. The latter is particularly attractive in a clinical setting because it would allow for simplified sampling with much smaller needles, lower morbidity and complication rates, better patient acceptance, thus obviating the need for anesthesia and monitored deep sedation.^[^ ^25^ ^]^ i2SCAN was designed to enable such analyses on scant cellular samples (10^2^–10^4^ cells) and may thus find particular use in serial immune cell profiling,^[^ ^10^ ^]^ global health applications,^[^ ^8^ ^]^ or whenever lesions are too small or central (e.g., endoscopy, fluid sampling) for core biopsies or resection.

i2SCAN integrates three technical advances into a single workflow. First, it is based on a compact, multichannel imaging system equipped with a lateral light illumination setup, a penta filter, and an RGB camera. The device has no moving filter parts, uses LEDs rather than lasers, and is thus fast and economical. Second, the system utilizes FAST‐reagents that allow fluorochrome modification of antibodies with subsequent ability to quench such fluorochromes in a spatially controlled way via click chemistry.^[^ ^9^ ^]^ We have directly programmed all of this into the linker and developed various iterations of this design.^[^ ^9^, ^26^, ^27^ ^]^ This enables immediate and complete quenching of the fluorescence once an image is acquired and subsequent cycles of staining can be performed rapidly. In the current study, we used quench times of 15 min for cells and 45 min for tissue sections. However, in other studies and using higher concentrations of Tz, we have used quench times as short as 10 min. Third, i2SCAN uses automated image analysis for spectral deconvolution and thresholding algorithms to compare expression data from single cells. Combined, the information can be used for clinical decision making, immunologic phenotyping, pathway analysis, or other relevant questions in human biology.

The depth of phenotyping is primarily limited by the antibody panel used. Here, we used a panel of 14 antibodies relevant to the differential diagnosis of breast lesions. Additional FAST panels are being developed for immune cell profiling of the tumor microenvironment^[^ ^10^ ^]^ and live‐cell imaging,^[^ ^9^ ^]^ while other panels have been developed for breast cancer pathway analysis.^[^ ^11^ ^]^ Additional panels are simple to add as long as high‐quality antibodies are available. Antibody modification with FAST reagents is straightforward and does not require deep chemical expertise. In the current study, we focused on five different fluorochrome channels and utilized the sixth channel (DAPI) to image all cellular nuclei across every cycle. This facilitated cell segmentation and registration, as the DAPI channel in each cycle served as a spatial alignment map. While we used a total of four cycles in the current study, we have expanded the concept to over 15 cycles in experimental samples. It is further possible to expand the diagnostic information content by combining markers to identify new unique cell populations. For example, a 20 marker panel can be used in a combinatorial fashion by combining multiple markers to identify hundreds of distinct cell populations.^[^ ^3^ ^]^

A key in the analytical pipeline is stringent quality control of antibodies (signal comparison to IgG control) and the use of quality control measures routinely employed in clinical immunofluorescence assays and flow cytometry designs.^[^ ^23^ ^]^ These include avoiding fluorochromes with spectral overlap, low quantum‐yield fluorochromes, environmentally sensitive fluorochromes, or antibodies with high background binding. It also includes proper signal compensation across channels, appropriate scaling and referencing to another gold standard (immunohistochemistry, proteomics), and judicious combinations of antibody panels, i.e., pairing bright fluorophores with dimly expressed markers. i2SCAN is compatible with use of fluorescent proteins, commercially available prelabeled antibodies, other labeling methods as well as the FAST cycling technology used here. Our results show that the RGB fingerprinting enabled accurate compensation for fluorescence bleed through between channels. RGB fingerprinting was possible in part by using a new color CMOS imager that matches the sensitivity of older monochromatic ones. We furthermore show that i2SCAN yields accurate information on marker expression on a single‐cell basis. However, care has to be taken to translate such information into actionable clinical diagnoses. Currently, such information is limited, and prospective trials will have to be conducted to determine the accuracy of new approaches. The current approach and previous iterations can potentially enable clinical diagnoses with 10^2^–10^4^ cells.^[^ ^8^ ^]^ This is much lower than the 10^3^–10^5^ cells required for traditional analyses. Irrespective of the findings and limitations, patterns of biomarkers identified in samples should always be validated by additional data.

Given the wealth of information provided by the i2SCAN workflow and its affordability, we anticipate that it could become a useful adjunct to diagnostics in the wider community.

## 4 Experimental Section

### i2SCAN System

Six LEDs were used for the imaging in brightfield and five fluorescence channels (six fluorochromes): a BF LED (Adafruit, 1622) for brightfield, an L385 (LuxeonStar, SZ‐05‐U3) with a low‐pass filter (400 nm, Edmund Optics, 84–689) for DAPI and BV605, an L470 (LuxeonStar, SZ‐05‐H3) with a 10 nm bandpass filter (470 nm central wavelength, Edmund Optics, 65–083) for AF488, an L567 (LuxeonStar, SZ‐05‐H9) with a 25 nm bandpass filter (550 nm central wavelength, Edmund Optics, 86–643) for AF555 or AF594, an L627 (LuxeonStar, Sz‐05‐H6) with a 10 nm bandpass filter (640 nm central wavelength, Edmund Optics, 65–107) for AF647, an L720 (LuxeonaStar, SP‐03‐D4) with a 25 nm bandpass filter (750 nm central wavelength, Edmund Optics, 86–647) for CF750. The L385 LED was fixed directly on the slide holder with a custom‐designed heat sink. The rest of LEDs were fixed on a custom‐designed holder; each LED module consisted of a focusing lens, an excitation filter, the LED, and a heat sink. The optical imaging module was consisted of an objective lens (Nikon, 20×, 0.70 NA), a penta filter (Semrock, FF01‐432/515/595/681/809‐25), and a 2448×2048 5‐MP color imaging sensor (TheImagingSource, DFM 37UX250‐ML containing a 2/3 inch Sony CMOS Pregius sensor, IMX250). A linear actuator (Dings’ Motion USA, Nema 8 Kaptive) was used to change the position of the imaging sensor for auto‐focusing (Figure S8, Supporting Information). The optomechanical components were either purchased from Thorlabs or designed in‐house and custom‐built through Xometry. A micro‐controller (Arduino, Arduino Nano) was used to control a hall‐effect sensor (Littelfuse, 55140–3H‐02‐A), the linear actuator and the LEDs, automatic focusing, and image acquisition, respectively. The CAD drawings for the custom‐designed slide holder and heat sinks are freely available for academic researchers at https://csb.mgh.harvard.edu/bme_software.

### User Interface

The control software with a graphical user interface was written in Visual Basic (Visual Studio 2019) and is freely available for academic researchers at https://csb.mgh.harvard.edu/bme_software. Daily calibration, auto‐focusing, sample imaging, image analysis, and report generation were all built into the software to facilitate end‐user operation. Daily calibration was performed using AccuCheckTM ERF reference particles (Thermo Fisher Scientific, A55950) to ensure that the detected fluorescence signals on respective channels were within the thresholds. The protocol for sample imaging was as follows:^[^ ^1^ ^]^ Sample slide was mounted on a holder and inserted into the system. The holder positioning was detected by the Hall sensor (Littelfuse, 55140–3H‐02‐A).^[^ ^2^ ^]^ Auto‐focusing was performed first through a coarse focal search in the DAPI channel; and then a focus quality assessment (FQA) was automatically performed by analyzing the focus quality within seven focal planes. To determine the best focus plane (Figure S8, Supporting Information), the imager was displaced in the axial direction by the linear actuator with a step size of 150 µm. A reference‐free FQA called FQPath^[^ ^28^ ^]^ was used to quantify the focus plane quality.^[^ ^3^ ^]^ Once the best focal plane was determined, the image capturing process started by switching on each LED for 5 s to stabilize its intensity, followed by image capture based on pre‐set imaging conditions (including exposure time and camera gain). The image capturing process was completed within 1 min.^[^ ^4^ ^]^ Image analysis was then performed including image alignment, cell‐nucleus segmentation, fingerprint analysis, and classification. The details are presented in the later section.^[5]^ The analysis report was generated. For daily calibration operation and focus analysis, Matlab dynamic‐link libraries were leveraged and integrated into the Visual Studio platform. Image analysis was done in Python and integrated through the Python extension.

### Cell Culture

A panel of breast cancer cell lines with different expression of triple markers (ER, PR, HER2) was used for assay validation: MCF‐7, BT474, HCC1954, and MDA‐MB‐231. All cell lines were purchased from the American Type Culture Collection (ATCC, Manassas, VA, USA). Human breast cancer cell lines MCF‐7 and MDA‐MB‐231 were maintained in Dulbecco's modified Eagle's medium (DMEM, Thermo Fisher Scientific, Waltham, MA, USA); BT474 and HCC1954 were maintained in RPMI 1640 (Thermo Fisher Scientific, Waltham, MA, USA). All media were supplemented with 10% fetal bovine serum (FBS, Bio‐Techne Sales) and 1% penicillin‐streptomycin (Thermo Fisher Scientific, Waltham, MA, USA) and cultured at 37 °C with 5% CO_2_. All cell lines were routinely tested using MycoAlert mycoplasma detection kit (Lonza, Basel, Switzerland). Peripheral blood mononuclear cells (PBMCs) were obtained from Stemcell Technologies (Cambridge, MA, USA).

### FAST Antibodies

FAST probes were built around a modular linker between fluorophores and antibodies with an embedded *trans‐*cyclooctene (TCO) for clicking with a tetrazine‐conjugated quencher. FAST probes were custom synthesized at large scale, stored as the carboxylic acids, and activated for antibody labeling with in situ activation chemistry.^[^ ^9^ ^]^ All reagents were obtained from commercial sources at the highest grade available and used without further purification. Fluorophores were purchased from Click Chemistry Tools or Fluoroprobes. BHQ‐3 Amine was purchased from LGC Biosearch Technologies. N‐α‐Boc‐N‐*ε*‐Fmoc‐Lysine was purchased from Chem‐Impex. Amino‐dPEGn‐carboxylic acids (*n* = 4,6) were obtained from Quanta BioDesign (Pain City, OH, USA). Dry solvents and coupling reagents were obtained from Sigma Aldrich (St. Louis, MO, USA).

Bovine serum albumin (BSA)‐free antibodies were purchased (Table 3) and then modified with FAST probes as described.^[^ ^9^ ^]^ Antibodies were exchanged into bicarbonate buffer (pH 8.4) using a 40k Zeba column (Thermo Fisher Scientific, Waltham, MA, USA). After buffer exchange, antibodies were incubated with a fivefold to tenfold molar excess of the Dye‐TCO‐NHS molecule (FAST probe) with 10% dimethyl sulfoxide for 25 min at room temperature (RT) protected from light. The conjugation reaction was loaded onto another 40k Zeba column equilibrated with phosphate‐buffered saline (PBS) for desalting and removal of unreacted FAST probes. To determine the degree of labeling, the absorbance spectrum of the FAST‐labeled antibody was measured using a Nanodrop 1000 (Thermo Fisher Scientific, Waltham, MA, USA), applying the known extinction coefficients of the dye, IgG antibody, and correction factor for the dye absorbance at 280 nm. The FAST‐labeled antibodies were stored in the dark at 4 ℃ in PBS until usage. All antibody conjugates were extensively validated prior to use as described elsewhere .^[^ ^10^ ^]^

### Immunostaining and Quenching of Cells

Cells were fixed for 15 min with CytoRich Red (Thermo Fisher Scientific, Waltham, MA, USA) and permeabilized for 10 min with BD perm/wash buffer (BD Biosciences, Franklin Lakes, NJ) prior to staining. After blocking with assay buffer supplemented with 2.5% BSA and 2.5% normal goat serum for 10 min, cells were stained with FAST‐conjugated antibodies for 20 min at RT in the dark. Stained cells were washed with PBS three times to remove unbound antibody before imaging. Following image acquisition, cells were briefly incubated with 10 × 10^−6^ m Tz‐BHQ (<10 min) in PBS‐bicarbonate buffer (pH 9) for quenching. Free Tz‐BHQ was removed by three washes with PBS, and the cells were imaged again in the same fields of view to record quenched signal. The same staining, imaging, and quenching cycle was repeated until all of the target proteins were imaged.

### Immunostaining and Quenching of FFPE Sections

Tissue sections were stained in similar fashion as described for cells. The paraffin‐embedded sections (tissue microarrays from US Biolab, Rockville, MD, USA; Sigma‐Aldrich, St. Louis, MO, USA; Stat Lab, McKinney, TX, USA; US Biomax, Rockville, MD, USA) were de‐paraffinized and rehydrated. Heat‐induced antigen retrieval was performed using Retrievagan A at pH 6 (BD Biosciences, Franklin Lakes, NJ) and the sections were permeabilized with 0.3% Triton X‐100 (Sigma‐Aldrich, St. Louis, MO, USA) in PBS for 10 min at RT. The tissue sections were blocked with intercept (PBS) blocking buffer (Licor) at RT for 1 h and FAST conjugated antibodies diluted in intercept (PBS) blocking buffer were incubated at RT. After washing the tissue with PBS, nuclei were counterstained with DAPI (Thermo Fisher Scientific, Waltham, MA, USA). The images were captured by using a digital scanner NanoZoomer 2.0RS (Hamamatsu, Japan), an automated fluorescence microscope, BX63 (Olympus, Tokyo, Japan), and i2SCAN. After imaging, the FAST conjugated antibodies were quenched with 0.01 × 10^−3^ m Tetrazine Quencher (BHQ3‐Tz) in 0.1 m sodium bicarbonate (NaHCO_3_) pH 9.0 at RT for 45 min and washed with PBS. 100 × 10^−6^ m TCO Blocker (dTCO‐PEG6) in PBS was applied to the tissue sections for 15 min at RT prior to the next FAST cycle staining. Immunohistochemistry (IHC) staining was performed on adjacent sections to validate FAST‐staining. Antibody staining was performed for 1 h at RT. To block endogenous peroxidase activity, 1% hydrogen peroxide was applied for 10 min at RT prior to the staining and VECTASTAIN ABC Kit (Vector Laboratories, Burlingame, CA, USA) and AEC (3‐amino‐9‐ethylcarbozole) substrate (Dako/Agilent, Santa Clara, CA, USA) were used for color development. The tissue sections were counterstained with Harris Hematoxylin (Sigma‐Aldrich, St. Louis, MO, USA).

### Flow Cytometry

Cell lines with different expression patterns of HER2, ER, and PR (BT474, HCC1954, and MDA‐MB‐231) and a 1:1 mixture of human PBMCs and cancer cell lines were analyzed by flow cytometry (LSRII, BD Biosciences, Franklin Lakes, NJ) to validate the results obtained by i2SCAN. PBMCs were purchased from Stem Cell Technologies. The same set of antibodies against HER2, ER, EGFR, EpCAM, and CD45 were used to stain the cells for flow cytometry and i2SCAN analysis.

### Image Analysis

All image analysis was done in Python. Briefly, images were aligned, segmented, and background subtracted before the average cellular fluorescence intensity was calculated. Camera noise was removed by subtracting a dark image acquired without a sample. To correct for pixel translations and rotation that occur in imaging between cycles, acquired images were aligned by a coordinate transform. The transform matrix was calculated with feature detection and matching using Oriented FAST and Rotated BRIEF (ORB) and Brute Force Hamming implemented in the OpenCV library.^[^ ^29^ ^]^ Nuclei and cells were segmented using Mesmer^[^ ^30^, ^31^ ^]^ or Cellpose^[^ ^32^ ^]^ from the maximum‐intensity‐profile of all cycles. Cells with more than 1 nucleus and a nuclear‐to‐cell size ratio greater than 1 were excluded from analysis. Quenched images from the previous cycle were subtracted from the stained image for background correction. For cycle 1, autofluorescence images were subtracted instead of a quenched image. The average fluorescence intensity was calculated for each identified cell. After segmentation, cells were spectrally unmixed and the average fluorescent intensity was calculated. Cells were normalized to the 10th percentile and the 99th percentile of the mean fluorescent intensity was used for analysis. Threshold levels for marker positivity were set as a mean intensity plus three standard deviations of control IgG samples. Processed images are quench‐subtracted and synthetic images show cell averages or phenotypes.

The fingerprint analysis was a pixel‐based linear unmixing method. It assumed that the total signal in each RGB sensor, *S*, was linearly proportional to the combination of contributing fluorochromes, *F*. The system of equations was solved with a linear least‐squares fit bounded by zero and has the following matrix form: *S* = *AF*.

The unmixing matrix *α* was experimentally calculated from single fluorochrome stains.

### Diagnostic Time

The overarching goal of this project was to develop a comprehensive workflow that could yield diagnostic information within a work day. The times were therefore optimized for i) tissue processing and staining, ii) imaging per field of view, and iii) image analysis. As a general rule, imaging was the fastest portion of the diagnostic time, taking less than 1 min per field of view (Figure S9, Supporting Information). Spectral unmixing was currently a slower part of the analysis (accounting for more than 98% of the computational time). The computational time for spectral unmixing could decrease by avoiding fluorochromes with spectral overlap and thoughtful assay design. It was also possible to speed up unmixing by using GPUs. Quench times were concentration dependent, with higher Tz concentrations quenching faster.

### Statistics

Statistical analyses and data plotting were performed in Python 3.7.0 and GraphPad Prism 9. Linear least‐squares fitting was performed for correlations between flow cytometry and i2SCAN with the Pearson correlation coefficient quantifying the correlations (two‐sided *p*‐value with *α* = 0.05). For image processing, quality control excluded segmented cells with more than 1 nucleus and a nuclear‐to‐cell size ratio greater than 1. To identify outliers, cells were normalized to the 10th percentile and the 99th percentile of the mean fluorescent intensity was used for analysis. Processed images were quench‐subtracted and synthetic images showed cell averages or phenotypes.

## Conflict of Interest

The authors declare no conflict of interest.

## Author Contributions

H.M.P., L.K.C., H.L., and H.I. contributed equally to the manuscript. System design and assembly: L.K.C., H.L., H.I., R.W. Software: L.K.C., H.M.P. Experiments and synthesis: H.M.P., L.K.C., J.O., Y.I., J.C.T.C. Data analysis: all authors. Writing: R.W. and all coauthors.

## Supporting information

Supporting Information

## Data Availability

The data that support the findings of this study are available from the corresponding author upon reasonable request.
