# Supplementary material for: Integrated Analytical System for Clinical Single‐Cell Analysis
Source: Adv Sci (Weinh). 2022 May 4;9(20):2200415. doi: 10.1002/advs.202200415 (PMC9284190; doi:10.1002/advs.202200415)

ADVANCED SCIENCE
Open Access

Supporting Information

for *Adv. Sci.*, DOI 10.1002/advs.202200415

Integrated Analytical System for Clinical Single-Cell Analysis

*Hannah M. Peterson, Lip Ket Chin, Yoshi Iwamoto, Juhyun Oh, Jonathan C. T. Carlson, Hakho Lee, Hyungsoon Im and Ralph Weissleder**

Supporting information

Integrated analytical system for clinical single cell analysis

Hannah M. Peterson[1†], Lip Ket Chin[1†], Yoshi Iwamoto[1], Juhyun Oh[1], Jonathan C.T. Carlson[1,2], Hakho Lee[1,3#], Hyungsoon Im[1,3#], Ralph Weissleder[1,2,3,4]*

[1] Center for Systems Biology, Massachusetts General Hospital, Boston, MA 02114, USA
[2] Cancer Center, Massachusetts General Hospital, Boston, MA 02114, USA
[3] Department of Radiology, Massachusetts General Hospital, Boston, MA 02114, USA
[4] Department of Systems Biology, Harvard Medical School, Boston, MA 02115, USA

†These authors contributed equally to the manuscript.
[#]These authors contributed equally to the manuscript.

*Corresponding author:
Ralph Weissleder
Center for Systems Biology
Massachusetts General Hospital
185 Cambridge St, CPZN 5206
Boston, MA, 02114
USA
1-617-726-8226
rweissleder@mgh.harvard.edu,

Keywords:

Cancer, Diagnostic, Biopsy, Deep Profiling

Fig. S1. Overview of clinical issues. Breast masses detected by palpation, mammography or other means are typically worked up by additional imaging, followed by biopsy or surgical excision depending on presentation, imaging findings and geographic location. Each of the follow-up approaches, while effective, has some shortcomings that are accentuated in different countries. To improve diagnostic accuracy of any intervention, shorten the time to diagnosis to same day and reduce cost, a highly multiplied tissues/FNA analysis system is required. Such as system and workflow were developed in this study.

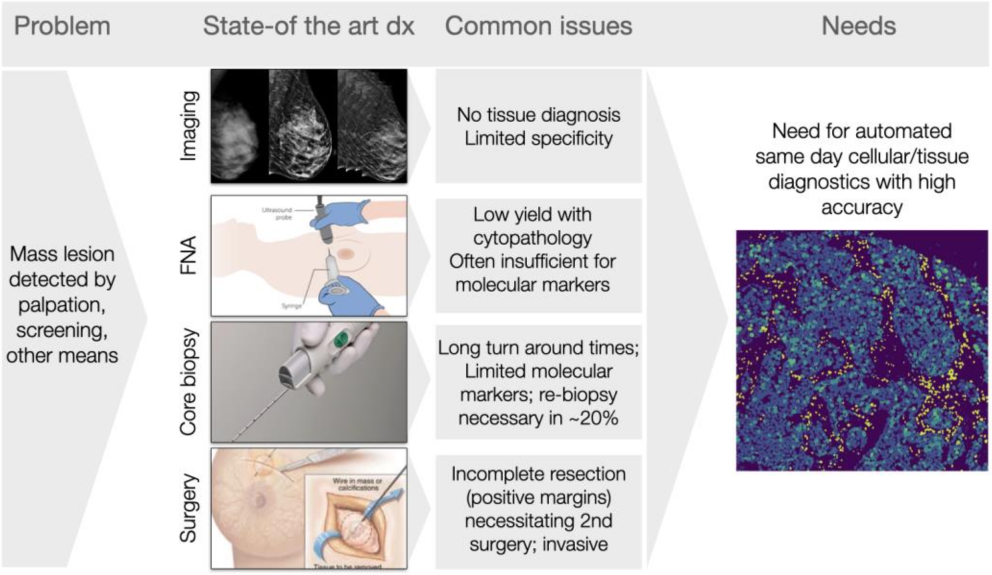

Fig. S2: i2SCAN system components. The integrated system measures 28 × 28 × 29 cm^3, and all optical components are contained within the enclosed device. There are 6 LEDs and no moving filter parts. The cost of retail components including the imaging sensor is currently approximately $5,000. Computational analysis is performed on a personal computer running Python.

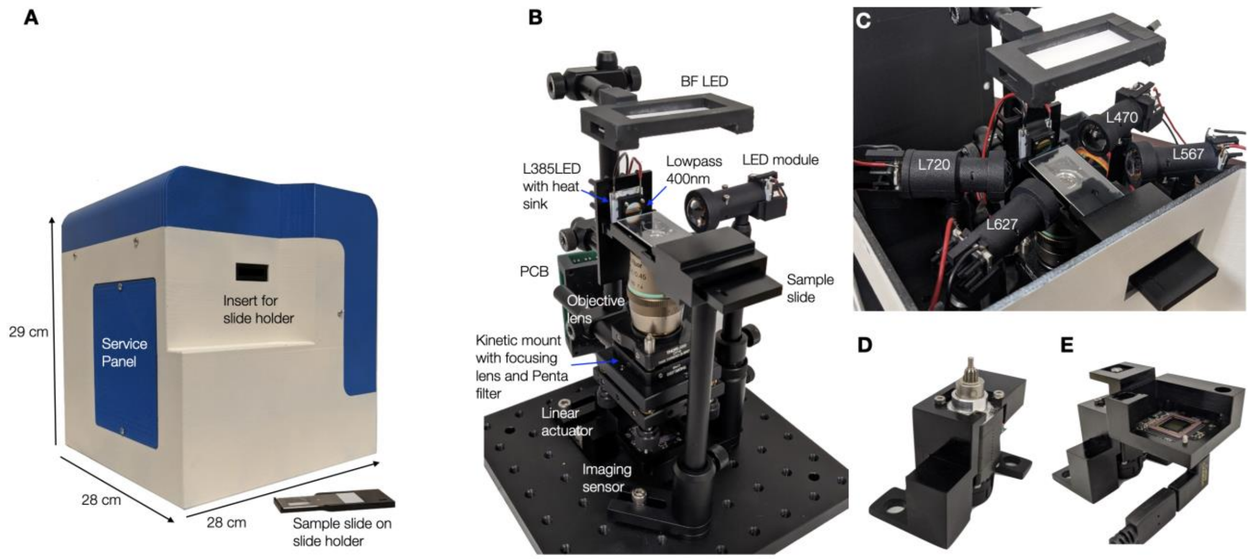

Fig. S3: Computational analysis of tissue sections
We redeveloped a computational pipeline for automated and integrated analysis of tissue sections. Shown are the different tasks and how they result in scaled synthetic images where biomarkers in single cells can be quantitated.

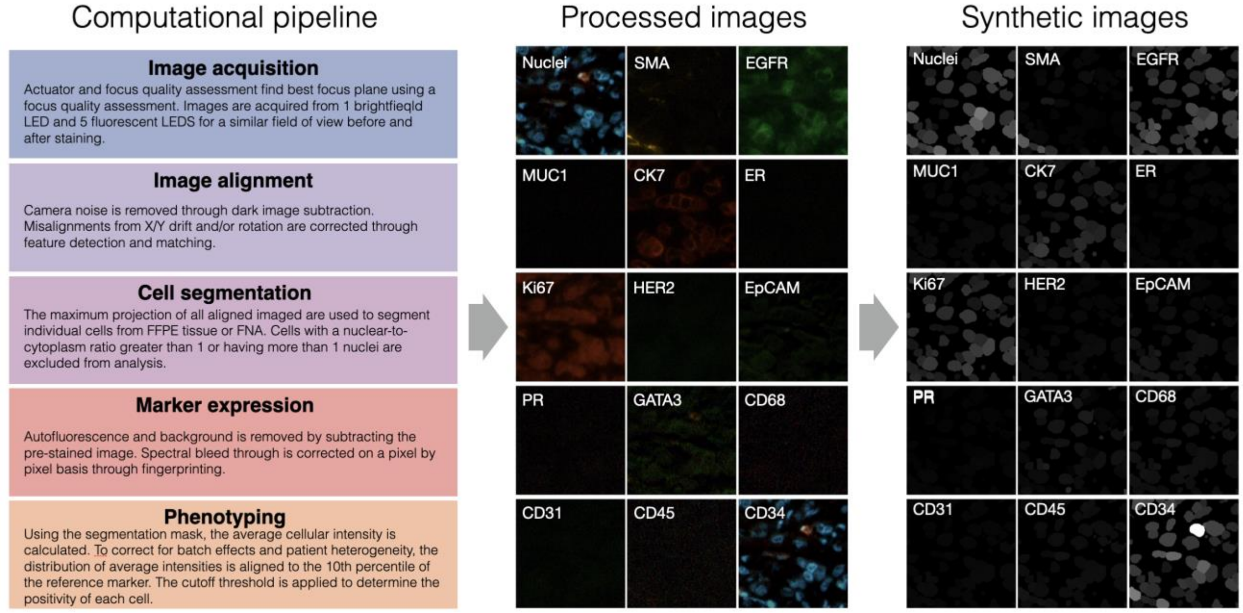

Fig. S4: Excitation and emission characteristics.
The i2SCAN system uses 5 LED with fixed bandpass filters for fluorescence excitation and an additional LED for bight light/colorimetric acquisition. The wavelengths for the penta emission filter are shown at the bottom.

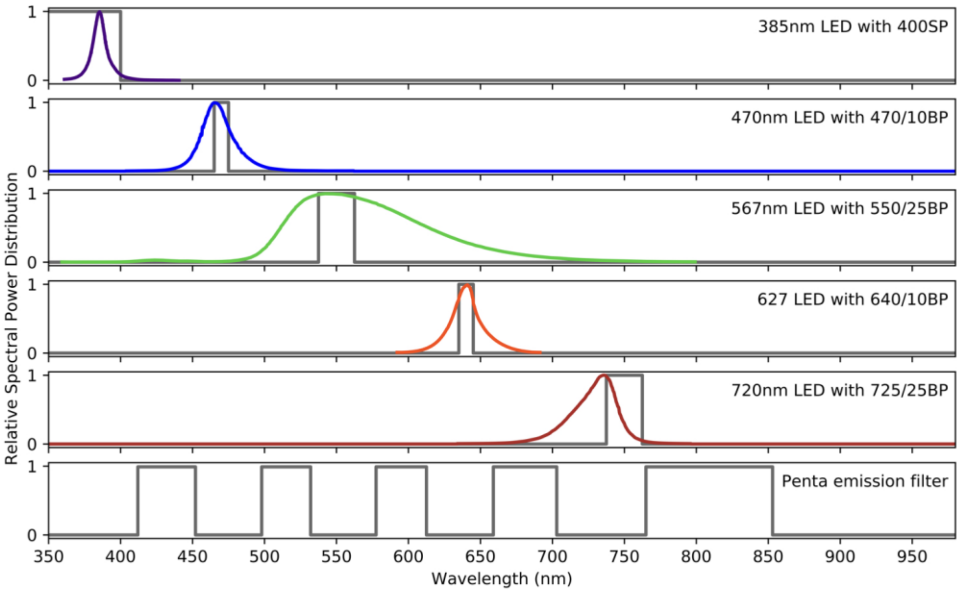

Fig. S5: Spectral unmixing in cells. HCC-1954 cells stained with different fluorochromes for HER2. Left panel shows representative examples of raw images. The right panel shows fingerprint corrected images. Note that DAPI and BV605 are separated and bleed through of CF750 is only present in one channel.

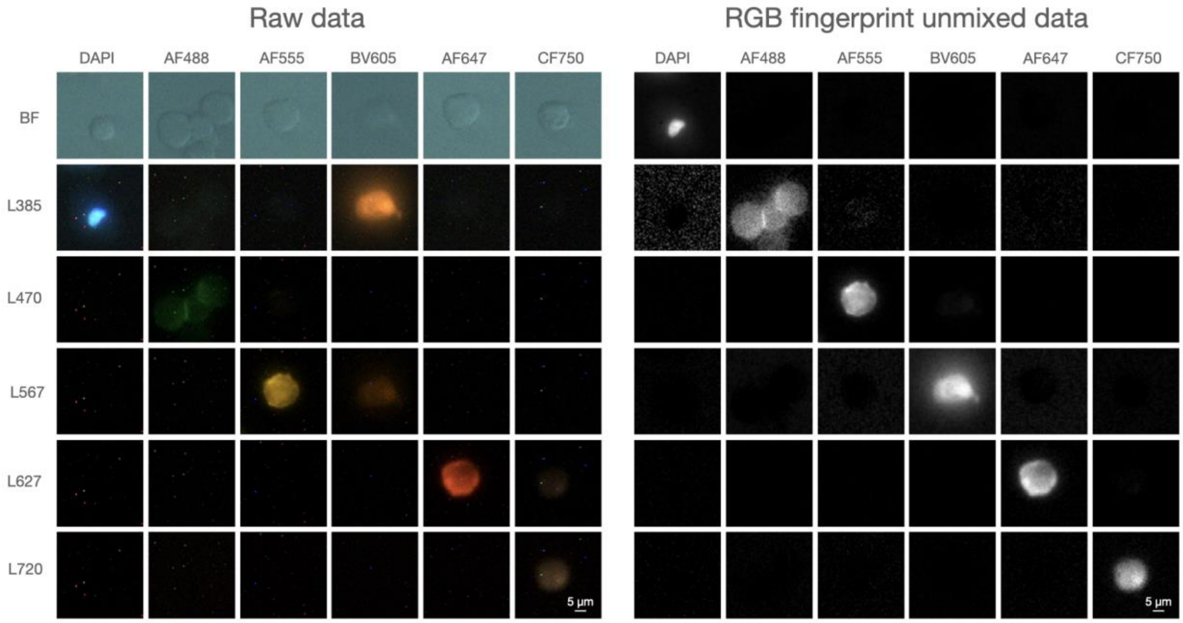

Figure S6. Overview of the chemistry enabling FAST imaging. The FAST linker synthesis starts with a lysine scaffold that contains a PEG_4 linker for efficient antibody conjugation: i) after TSTU activation (not shown), the protected Lysine succinimidyl ester was reacted with H_2N-PEG_4-CO_2H, then ii) deprotected with DCM/TFA (20%); iii) reaction with rTCO-PNP was followed by iv) removal of the Fmoc group with piperidine (7.5%). The completed linker was then functionalized with AF488, AF555 or AF647. To generate the quencher, BHQ3-amine was coupled with HTz-PEG_5-NHS to yield BHQ3-Tz in one step. The fluorescent signal of FAST-labeled antibodies can be efficiently quenched (>95%) with 10 µM of BHQ3-Tz in < 1 min (*9*).

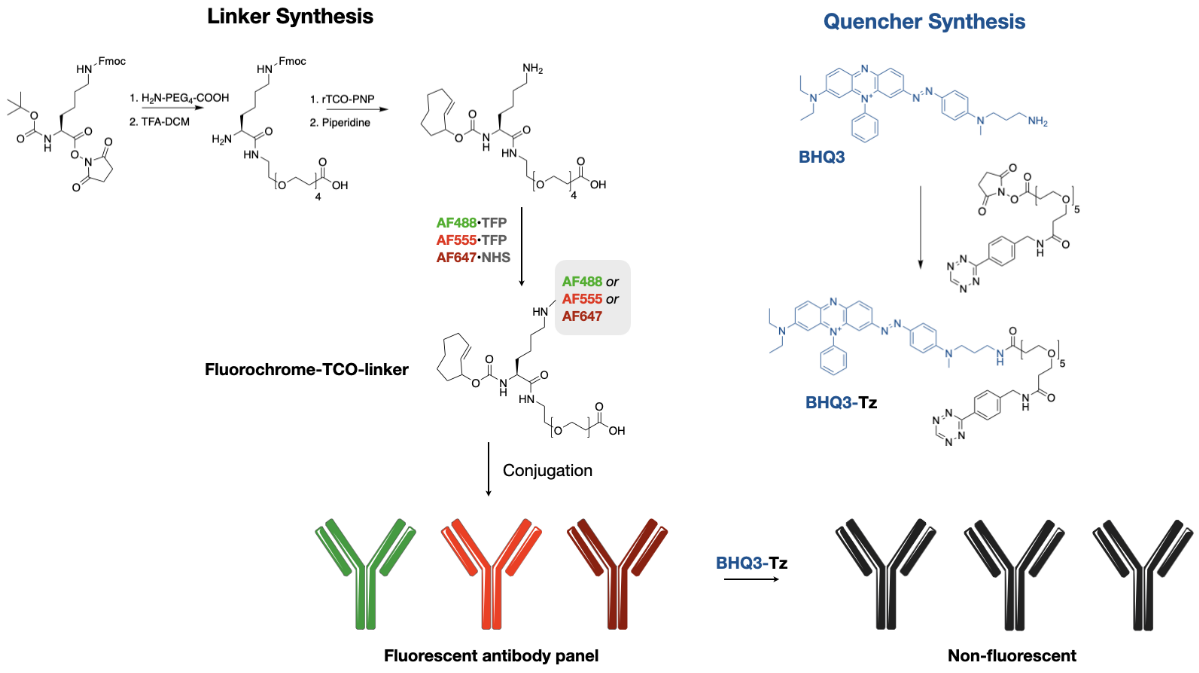

Fig. S7: Validation and comparison of imaging quality. A. To quantitatively compare image quality of FAST-stained tissue sections we obtained images using i2CSAN and a conventional fluorescence microscope (BX63, Olympus, Tokyo, Japan). Shown here is the Pearson correlation (Pearson *r* = 0.92) between i2SCAN and BX63 images for TROP2 expression in a breast cancer slide. **B.** Visual comparison of FAST-stained EGFR expression in a breast cancer tissue microarray, images captured with i2SCAN, a conventional fluorescence microscope (BX63, Olympus, Tokyo, Japan), and a slide scanner (NanoZoomer 2.0RS, Hamamatsu, Japan).

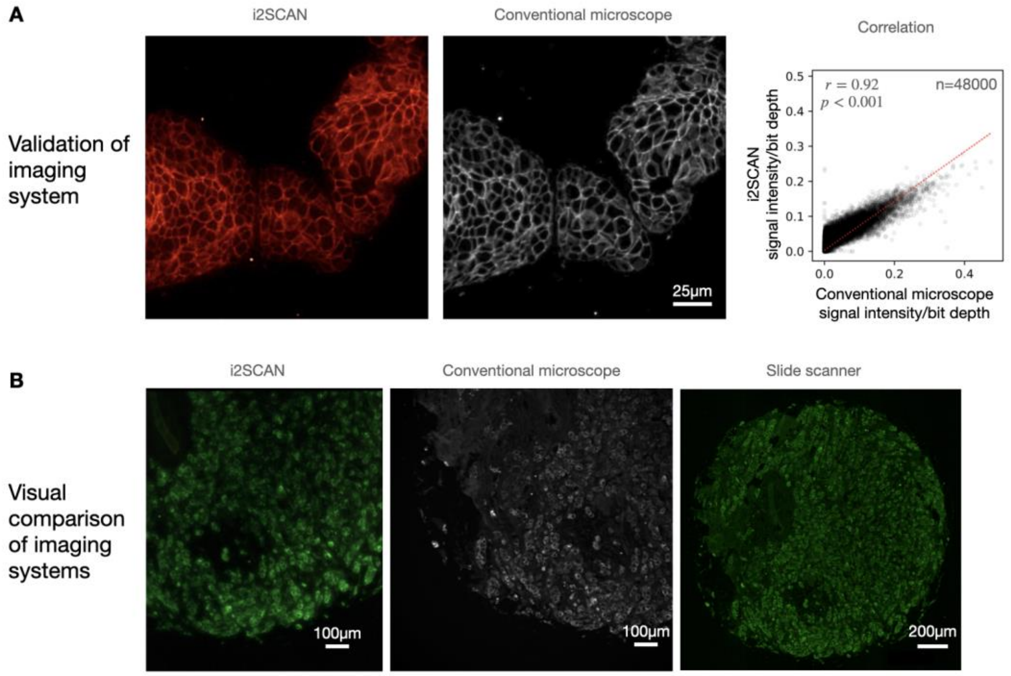

Fig. S8: Autofocus. The i2SCAN system has an auto-focus feature to improve image quality. Left: The FQPath metric ***(28)*** is calculated for images obtained at 150µm intervals and plotted. The lowest FQPath score (FQPath=5.37 in this example) is the one with the image in focus. Right: examples of raw cellular images obtained at different z-intervals.

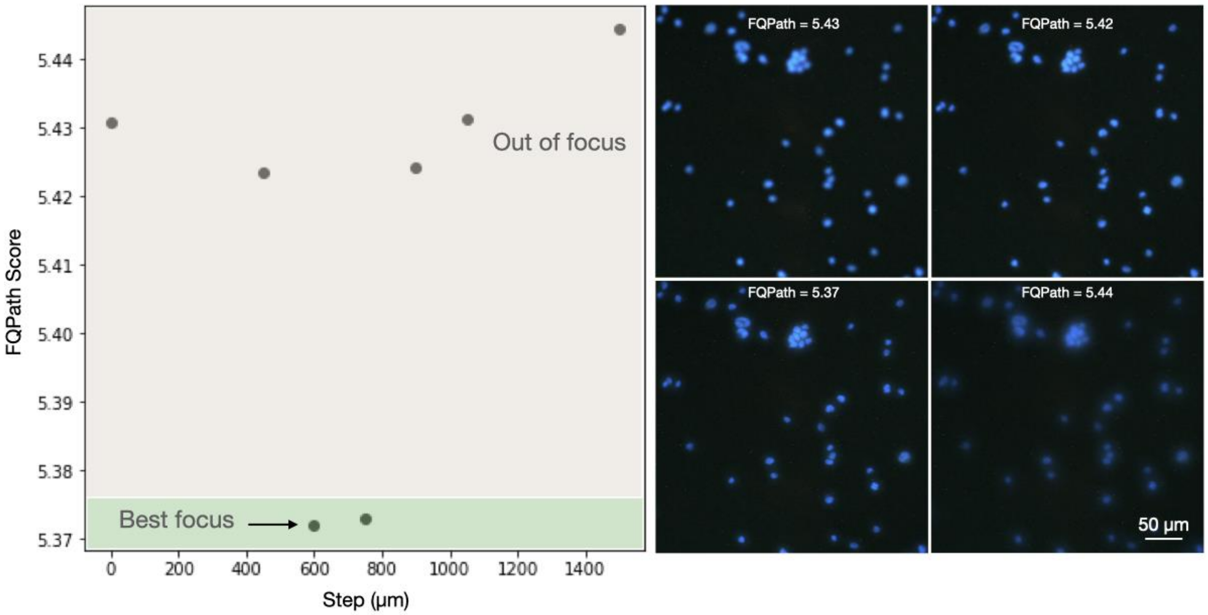

Fig. S9: Overview of steps in image acquisitions and time lines. The overarching goal was to develop a workflow for same day processing. Note that the actual imaging is fast. The majority of time is used for cycling.

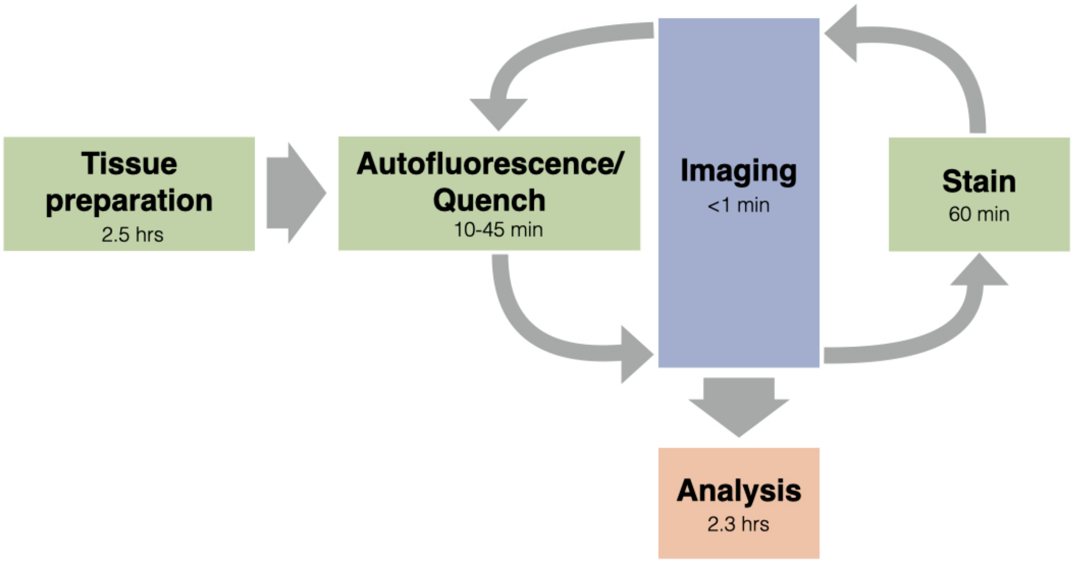

Supplement: Supplementary file 1 — Supporting Information [file ADVS-9-2200415-s001.pdf]
